# Machine learning for epithelial ovarian cancer platinum resistance recurrence identification using routine clinical data

**DOI:** 10.3389/fonc.2024.1457294

**Published:** 2024-11-08

**Authors:** Li-Rong Yang, Mei Yang, Liu-Lin Chen, Yong-Lin Shen, Yuan He, Zong-Ting Meng, Wan-Qi Wang, Feng Li, Zhi-Jin Liu, Lin-Hui Li, Yu-Feng Wang, Xin-Lei Luo

**Affiliations:** ^1^ Hematology Oncology Department, the Southern Central Hospital of Yunnan Province, Honghe, Yunnan, China; ^2^ Geriatric Oncology Department, the Third Affiliated Hospital of Kunming Medical University, Kunming, Yunnan, China; ^3^ Department of Oncology, the Pingxiang People’s Hospital, Pingxiang, Jiangxi, China; ^4^ Department of Oncology, The First Hospital of Nanchang, Nangchang, Jiangxi, China; ^5^ Department of Spinal Surgery, Southern Central Hospital of Yunnan Province, Honghe, China

**Keywords:** platinum resistance, recurrence, model, nomogram, early detection of cancer

## Abstract

**Background:**

Most epithelial ovarian cancer (EOC) eventually develops recurrence. Identification of high-risk patients can prompt earlier intervention and improve long-term outcomes. We used laboratory and clinical data to create models based on machine learning for EOC platinum resistance recurrence identification.

**Methods:**

This study was designed as a retrospective cohort analysis. Initially, we identified 1,392 patients diagnosed with epithelial ovarian cancer who underwent platinum-based chemotherapy at Yunnan Cancer Hospital between January 1, 2012, and June 30, 2022. We collected data on the patients’ clinicopathologic characteristics, routine laboratory results, surgical information, details of chemotherapy regimens, and survival outcomes. Subsequently, to identify relevant variables influencing the recurrence of platinum resistance, we screened thirty potential factors using two distinct variable selection methods: Lasso regression and multiple logistic regression analysis. Following this screening process, five machine learning algorithms were employed to develop predictive models based on the selected variables. These included decision tree analysis (DTA), K-Nearest neighbor (KNN), support vector machine (SVM), random forest (RF), and eXtreme gradient boosting (XGBoost). The performance of these models was compared against that of traditional logistic regression. To ensure robust internal validation and facilitate comparison among model performance metrics, a five-fold cross-validation method was implemented. Key performance indicators for the models included the area under the receiver operating characteristic curve (AUC), sensitivity, specificity, and average accuracy. Finally, we will visualize these models through nomograms, decision tree diagrams, variable importance plots, etc., to assist clinicians in their practice.

**Results:**

Multiple logistic regression analysis identified eight variables associated with platinum resistance recurrence. In the lasso regression, seven variables were selected. Based on the findings from both Lasso regression and multiple logistic regression analysis, models were developed using these 7 and 8 factors. Among these, the XGBoost model derived from multiple logistic regression exhibited superior performance and demonstrated good discrimination during internal validation, achieving an AUC of 0.784, a sensitivity of 0.735, a specificity of 0.713, an average accuracy of 80.4%, with a cut-off value set at 0.240. Conversely, the LR model based on lasso regression yielded commendable results as well; it achieved an AUC of 0.738, a sensitivity of 0.541, a specificity of 0.836, with a cut-off value established at 0.154 and an accuracy rate of 79.6%. Finally, we visualized both models through nomograms to illustrate the significance of each variable involved in their development.

**Conclusions:**

We have successfully developed predictive models for platinum-resistant recurrence of epithelial ovarian cancer, utilizing routine clinical and laboratory data. Among these models, the XGBoost model—derived from variables selected through multiple logistic regression—demonstrated the best performance. It exhibited high AUC values and average accuracy during internal validation, making it a recommended tool for clinical use. However, due to variations in time and context, influencing factors may change over time; thus, continuous evolution of the model is necessary. We propose a framework for this ongoing model adaptation.

## 1 Background

Seventy percent of patients diagnosed with epithelial ovarian cancer (EOC) present at an advanced stage (Federation of International of Gynecologists and Obstetricians (FIGO) stages III and IV) (1–3) The standard treatment approach involves primary debulking surgery (PDS), aimed a t achieving no visible residual tumor, followed by adjuvant chemotherapy based on platinum and paclitaxel (4, 5). A significant proportion of patients can attain complete remission. However, approximately 75% of those with advanced-stage disease will ultimately experience a relapse, resulting in poor survival outcomes (6, 7).

Following first-line treatment, around 15% of patients exhibit platinum-resistant recurrence; conversely, many remain platinum-sensitive at the time of their initial recurrence. Nevertheless, after undergoing multiple relapses, most cases of advanced ovarian cancer inevitably progress to a state that is resistant to platinum-based therapies.

Treatment options for patients experiencing platinum-resistant recurrence are currently quite limited. Existing guidelines primarily advocate for non-platinum monotherapy in the management of platinum-resistant ovarian cancer. Although recent guidelines have introduced new combinations—such as oral cyclophosphamide combined with pembrolizumab and bevacizumab, fam-trastuzumab deruxtecan-nxki, and Mirevtuxiamab Soratansine plus bevacizumab—the overall efficacy for treating platinum-resistant ovarian cancer remains suboptimal (8). Effective methods or drugs capable of truly reversing resistance are scarce. The prognosis for patients with platinum-resistant recurrence is poor, characterized by a progression-free survival (PFS) time of only 3 to 4 months and a response rate to chemotherapy of less than 15%. The median survival duration is reported to be under 12 months (9).

Platinum-resistant recurrence is currently defined as ovarian cancer that responds to initial chemotherapy but progresses or relapses within six months following the completion of treatment. Beyond this six-month period, it is unlikely that patients will exhibit significant symptoms or signs indicative of recurrence. Recurrence is primarily assessed through imaging examinations and monitoring serum levels of Carbohydrate Antigen 125 (CA125). This necessitates waiting for disease progression before determining whether a patient has experienced a platinum-resistant relapse. If we can identify platinum-resistant recurrences early on, patients likely to be resistant may consider undergoing platinum-based treatments either as monotherapy or in combination with other agents during first-line therapy. For instance, the antiangiogenic agent bevacizumab can be effectively combined with poly (ADP-ribose) polymerase (PARP) inhibitors such as Olaparib as part of maintenance therapy, alongside appropriate applications involving cell cycle regulators (10–12). The initial chemotherapy cycle may be appropriately intensified, or conventional intraperitoneal perfusion chemotherapy should be considered to extend the platinum-free interval (PFI) and convert potential platinum-resistant patients into those who are platinum-sensitive. Furthermore, clinicians can modify the follow-up plan for patients to enable more rigorous surveillance for recurrence. Early detection of platinum-resistant recurrence empowers clinicians to reassess treatment strategies and individualize follow-up plans, thereby enhancing the long-term prognosis of ovarian cancer.

However, accurately predicting the recurrence of platinum resistance remains a significant challenge. Most risk models have been developed primarily to forecast

PFS and overall survival (OS) in ovarian cancer (13–15). Previous models assessing platinum sensitivity were constructed using logistic regression (LR), a conventional statistical approach (16). The performance of these models tends to decline when applied to populations outside the original study cohort. With the advent of machine learning and its expanding applications, researchers are increasingly exploring the use of artificial intelligence within the medical domain (17, 18). To enhance long-term prognostic outcomes for patients with EOC, we aim to utilize routine clinical and laboratory data to develop machine learning models that predict the recurrence of platinum resistance in EOC patients.

## 2 Methods

### 2.1 Study population

Participants included patients with EOC who received first-line treatment at Yunnan Cancer Hospital between January 1, 2012, and June 30, 2022. The following inclusion and exclusion criteria were established:

#### 2.1.1 Inclusion criteria

1. Surgical procedures were performed at our hospital, with a pathological diagnosis of EOC;
2. Administration of platinum-based first-line chemotherapy;
3. Availability of demographic information, clinical data, and follow-up records.

#### 2.1.2 Exclusion criteria

1. Patients who did not receive platinum-based neoadjuvant chemotherapy or platinum-based first-line chemotherapy;
2. Presence of multiple primary malignant tumors;
3. Undergoing other treatments such as maintenance therapy involving bevacizumab or PARP inhibitors;
4. Loss to follow-up prior to six months post-treatment initiation;
5. Enrollment period less than six months without recurrence occurring;
6. Diagnosis of severe infectious diseases or mental disorders.

### 2.2 Data collection

After reviewing the existing literature and consulting with experts, we identified the variables to be collected (16, 19–22). In accordance with the Transparent Reporting of a Multivariable Prediction Model for Individual Prognosis or Diagnosis statement (23), it was established that the number of outcome events in the development cohort should be at least ten times greater than the number of variables. Furthermore, we stipulated that each variable must have an event count exceeding ten; otherwise, it would be excluded from analysis. Concurrently, any missing values were addressed by removing cases where a variable was absent in more than 30% of patients.

Ultimately, we compiled a total of 30 variables encompassing sociodemographic characteristics, surgical records, chemotherapy-related information, routine laboratory tests—including complete blood count (CBC) values—as well as renal and liver function indicators and other chemotherapy-related metrics (see **Table 1**). Laboratory data such as CA125 levels, CBC results, and lactate dehydrogenase (LDH) serum concentrations were obtained within one week prior to the commencement of surgery. All relevant data were extracted from pathology reports and medical records.

**Table 1:** Clinicopathologic characteristics between platinum-resistant group and platinum- sensitive group.

Characteristics		All (1392)	Platinum-Resistant (294,%)	Platinum-Sensitive (1098,%)	P
Age (years)		51.08 ± 9.28	52.56 ± 8.48	50.69 ± 9.44	0.002
Histologic type					0.130
	Serous	947 (68.0)	224 (76.2)	723 (65.8)	
	Mucous	67 (4.8)	8 (2.7)	59 (5.4)	
	Endometrial	92 (6.6)	11 (3.7)	81 (7.4)	
	Clear cell	119 (8.5)	20 (6.8)	99 (9.0)	
	Others	167 (12)	31 (10.5)	136 (12.4)	
FIGO stage					<0.001
	I	240 (17.2)	12 (4.1)	228 (20.8)	
	II	141 (10.1)	12 (4.1)	129 (11.7)	
	III	747 (53.7)	196 (66.7)	551 (50.2)	
	IV	264 (19.0)	74 (25.2)	190 (17.3)	
Neutrophil count		5.1 ± 2.4	7.3 ± 2.2	7.2 ± 2.6	0.326
Monocyte count		0.4 ± 0.2	0.4 ± 0.3	0.4 ± 0.2	0.019
Hemoglobin (g/L)		129.19 ± 15.75	127.11 ± 14.73	129.75 ± 15.97	0.011
Platelet (10^9^/L)					<0.001
	<134.5*10^9/L	613 (44.0)	87 (29.6)	526(47.9)	
	≥134.5*10^9/L	779 (56.0)	207 (70.4)	572(52.1)	
Albumin		41.5 ± 7.0	39.7 ± 6.3	41.9 ± 7.1	<0.001
Prognostic nutritional index		63.0 ± 28.0	55.5 ± 23.9	65.0 ± 28.7	<0.001
LDH (U/L)		285.95 ± 362.12	362.97 ± 701.50	265.33 ± 181.16	<0.001
Ln (CA-125) (IU/mL)		6.27 ± 1.77	6.85 ± 1.55	6.11 ± 1.78	<0.001
Primary treatment strategy					<0.001
	PDS	904 (64.9)	142 (48.3)	762 (69.4)	
	NAC	488 (35.1)	152 (51.7)	336 (30.6)	
Supraclavicular lymph node metastasis					0.001
	No	1348 (96.8)	276 (93.9)	1072 (97.6)	
	Yes	44(3.2)	18 (6.1)	26 (22.4)	
Pleural effusion					0.024
	No	1019(73.2)	200(68.0)	819(74.6)	
	Yes	373(26.8)	94(32.0)	279(25.4)	
Peritoneal effusion					<0.001
	No	418(30.0)	60(20.4)	358(32.6)	
	Yes	974(70.0)	234(79.6)	740(67.4)	
Malignant ascites					<0.001
	No	871(62.6)	146(49.7)	725(66)	
	Yes	521(37.4)	148(50.3)	373(34)	
Appendix					<0.001
	No involvement	1125(80.8)	195(66.3)	930(84.7)	
	Yes	267(19.2)	99(33.7)	168(15.3)	
Upper abdominal surgery					<0.001
	No	1140(81.9)	213(72.4)	927(84.4)	
	Yes	252(18.1)	81(24.6)	171(15.6)	
Colon except for rectosigmoid colon					<0.001
	No involvement	1202 (86.4)	224(76.5)	978 (89.1)	
	Yes	189 (13.6)	69(23.5)	120 (10.9)	
Omentum (cm)					<0.001
	No	743	97 (53.4)	646 (58.8)	
	≤2	376	67 (19.8)	209 (19.0)	
	>2	373	130 (26.8)	243 (22.1)	
Diaphragmatic top					<0.001
	No involvement	875 (62.9)	125 (42.5)	750 (68.3)	
	Yes	517 (37.1)	169 (57.5)	348 (31.7)	
Liver surface					<0.001
	No involvement	1215 (87.3)	246 (83.7)	969 (88.3)	
	Yes	177 (12.7)	48 (16.3)	129 (11.7)	
Liver parenchyma					<0.001
	No involvement	1353(97.2)	280(95.2)	1703(97.7)	
	Yes	39(2.8)	14(4.8)	85(2.3)	
Spleen					<0.001
	No involvement	1303(93.6)	263(89.5)	1040(94.7)	
	Yes	89(6.4)	31(10.5)	58(5.3)	
Small bowel and mesentery					<0.001
	No involvement	894 (64.2)	136 (46.3)	758 (69.0)	
	Yes	498 (35.8)	158 (53.7)	340 (31.0)	
Pelvic floor tissue					<0.001
	No involvement	987(70.9)	183(62.2)	804(73.2)	
	Yes	405(29.1)	111(37.8)	294(26.8)	
Bladder					<0.001
	No involvement	985(70.8)	164(55.8)	821(74.8)	
	Yes	407(29.2)	130(44.2)	277(25.2)	
Residual tumor(cm)					<0.001
	<1	955(68.6)	137 (46.6)	818 (74.5)	
	≥1	437(31.4)	157 (53.4)	280 (25.5)	
Chemotherapy cycle					<0.001
	<6	322(23.1)	92 (31.4)	230 (20.9)	
	≥6	1070 (76.9)	202 (68.7)	868 (79.1)	
Types of platinum					<0.001
	Carboplatin	1291(92.7)	251 (85.4)	1040 (94.7)	
	Cisplatin or others	101(7.3)	43 (14.6)	58 (5.3)	

This study conducted follow-up visits every two cycles of chemotherapy through clinical assessments and radiological evaluations. The follow-up period spanned from April 2012 to December 2022.

Hydrothorax and ascites were defined as the presence of any pleural effusion or pelvic fluid detected via ultrasound. In patients with measurable tumors, recurrence was determined using the Response Evaluation Criteria in Solid Tumors (RECIST) based on CT scans. Recurrence was indicated by an increase of at least 20% in the sum of the maximum diameters of tumor lesions, along with an absolute increase of at least 5 mm, or by the emergence of new tumor lesions. For cases where cancer could not be measured, tumor recurrence was evaluated through serum CA125 levels: specifically, if a patient’s serum CA125 level exceeded the upper limit of the reference range on two separate occasions at least one week apart.

According to statements from the Gynecologic Cancer Oncology Group (CGOG), The time interval between completion of platinum-based chemotherapy and disease progression is referred to as the platinum-free interval (9). Platinum resistance was classified as occurring when PFI was less than six months. Conversely, patients with a PFI equal to or greater than six months—regardless of disease recurrence status—were categorized as part of a platinum-sensitive cohort (24).

This research received approval from the Ethics Committee of Yunnan Cancer Hospital.

### 2.3 Data analysis

An exploratory analysis was performed. Skewed distribution factors, such as serum CA-125 levels, were ln-transformed to address the skewness in the data. The distribution of continuous variables was presented using mean and standard deviation. The maximum Youden index was employed to identify the optimal cut-off point for continuous variables. For binary, continuous, and ranked data, we utilized the chi-square test, Student’s t-test, and Wilcoxon rank-sum test respectively.

### 2.4 Variable selection and model development

To select appropriate variables, we employed two methods for variable screening. Firstly, a univariate and multiple logistic regression analysis based on Akaike’s Information Criterion was utilized to identify variables predictive of platinum resistance. The second method involved lasso regression, which effectively eliminates factors with low contributions to the model’s predictive ability among highly collinear variables, thereby achieving the goal of reducing the number of variables.

**Figure 1:**
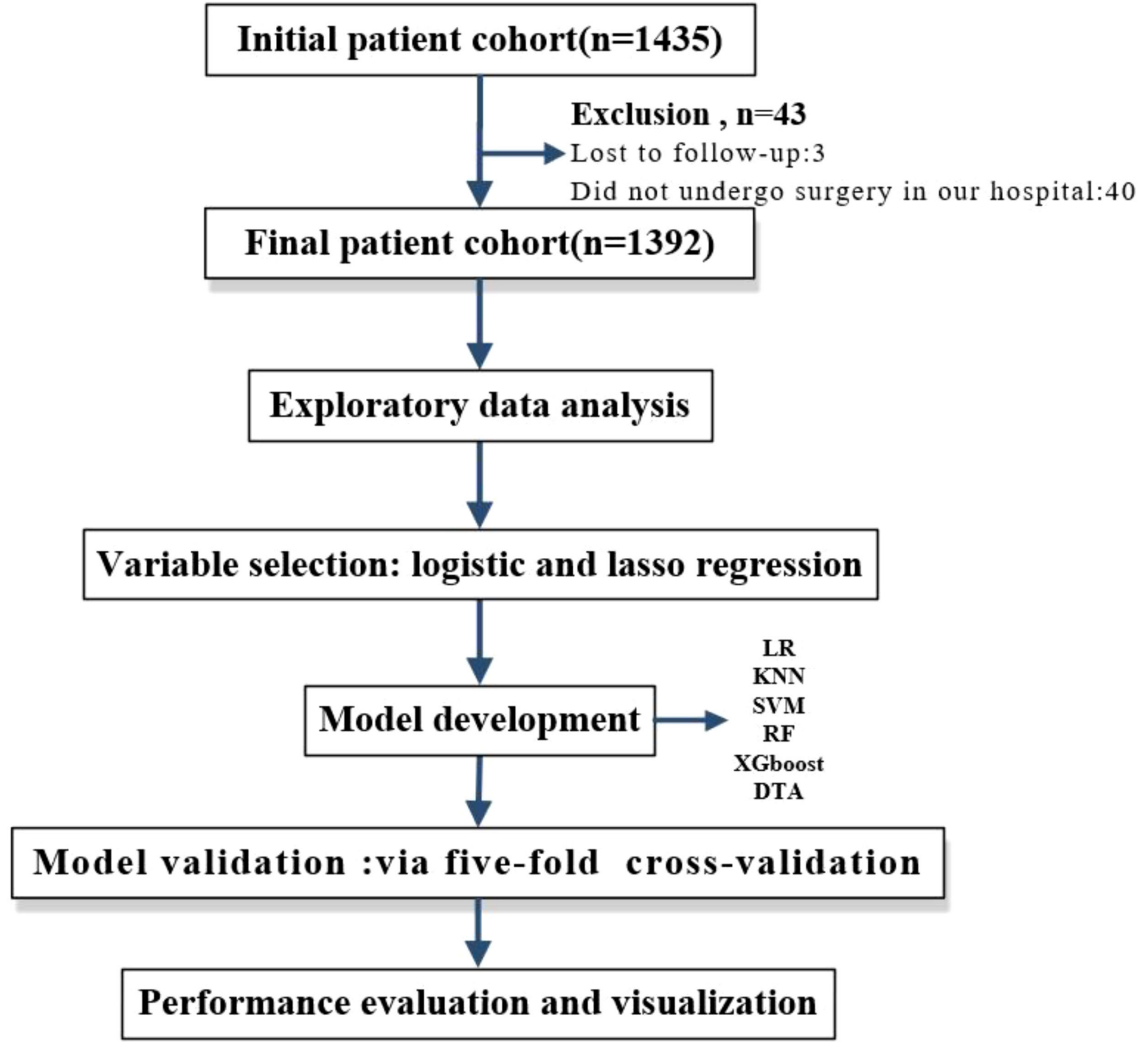
Overall workflow of statistical analysis.

In this study, five types of supervised machine learning classifiers were used to build models: decision tree analysis (DTA), support vector machine (SVM) (24, 25), K-Nearest neighbor (KNN), random forest (RF), and eXtreme gradient boosting (XGBoost). The traditional development method of LR served as the baseline for comparison. Performance indicators for the models included AUC, sensitivity, specificity, and average accuracy. Five-fold cross-validation was implemented to compare the performance across these six models (**Figure 1**). Exploratory data analysis was conducted using IBM SPSS Statistics software while all other statistical analyses were performed using R version 4.2.1. A p-value of less than 0.05 was considered statistically significant.

## 3 Results

### 3.1 Baseline information

A total of 1,435 patients were enrolled in the study; however, 40 patients did not undergo surgery at our hospital. The longest follow-up period was 118 months, while the shortest was 0 months, resulting in a median follow-up time of 13 months. Three cases were lost to follow-up (loss rate: 0.2%), leaving a final cohort of 1,392 patients for analysis. Among these, 294 patients (21.1%) experienced recurrence with platinum resistance (platinum-resistant group), whereas the remaining 1,098 patients were classified as platinum-sensitive.

Overall, significant differences between the two groups were observed concerning hemoglobin levels, platelet counts, and albumin concentrations (**Table 1**). In terms of primary treatment modalities, 904 patients (64.9%) underwent PDS, while 488 patients (35.1%) received interval debulking surgery following neoadjuvant chemotherapy (NAC). Notably, the platinum-sensitive group was younger on average compared to the platinum-resistant group (mean age: 50.69 vs. 52.56 years; p =0.002) and exhibited a higher likelihood of being in FIGO stages I-II.

Additionally, serum CA-125 levels were lower in the platinum-sensitive group compared to their resistant counterparts (mean: 2.65 vs. 2.97 IU/mL; p<0.001), as well as a reduced proportion receiving NAC treatment (30.6% vs.51.7%; p<0.001). Post-surgery outcomes indicated that complete cytoreduction rates in the platinum-sensitive group surpassed those seen in the platinum-resistant group significantly (74.5% vs.46.6%; P <0.001). While first-line chemotherapy regimens for both groups were comparable, the number of chemotherapy cycles administered to the platinum-resistant group was notably fewer than that given to their sensitive counterparts (p<0.001).

### 3.2 Model development

For patients whose pathological reports indicated adenocarcinoma without further specification to serous or mucinous types, the pathological classification was categorized as “other.” Consequently, the variable “histologic type” was excluded from the variable screening step.

Multivariate logistic regression analysis identified eight independent variables influencing platinum resistance recurrence: LDH levels, FIGO stage, platelet count, supraclavicular lymph node metastasis, primary treatment strategy, residual tumor size, type of platinum (carboplatin/cisplatin or others), and number of chemotherapy cycles (**Table 2**). Intuitively, involvement of the omentum and larger residual tumor size were associated with an increased risk of platinum-resistant recurrence; conversely, a greater number of chemotherapy cycles correlated with a reduced risk.

**Table 2:** Univariate and multivariate logistic regression analysis of independent risk factors affecting platinum resistance recurrence.

	univariate analysis		multivariate analysis	
Variable	OR (95%CI)	P	OR (95%CI)	P
FIGO stage				
I	–	–	–	–
II	1.767 (0.772-4.048)	0.178	1.805 (0.753-4.324)	0.184
III	6.759 (3.698-12.351)	<0.001	3.317 (1.628-6.757)	0.001
IV	7.400 (3.903-14.028)	<0.001	2.873 (1.328-6.215)	0.007
Age (years)	0.822 (0.563-1.200)	0.309	–	–
Neutrophil count	0.596 (0.461-0.770)	0.000	0.848 (0.148-4.863)	0.853
Monocyte count	1.669(1.051-2.650)	0.030	1.054 (0.621-1.788)	0.845
Hemoglobin (g/L)	0.990 (0.982-0.998)	0.011	1.000 (0.990-1.010)	0.092
Platelet (10^9^/L)	2.188 (1.659-2.886)	<0.001	1.532 (1.117-2.102)	0.008
Albumin (g/L)	0.957 (0.939-0.974)	<0.001	0.993 (0.969-1.017)	0.538
PIN	0.987 (0.982-0.992)	<0.001	1.000 (0.959-1.044)	0.994
LDH (U/L)	1.001 (1.000-1.002)	0.001	1.001 (1.000-1.001)	0.022
CA125	1.000(1.000-1.000)	0.005	1.000 (1.000-1.000)	0.691
Supraclavicular lymph node metastasis	2.689 (1.453-4.975)	<0.001	2.712 (1.319-5.573)	0.007
Pleural effusion	1.380 (1.042-1.826)	0.024	0.875 (0.642-1.226)	0.437
Peritoneal effusion	1.887 (1.383-2.574)	<0.001	0.972 (0.669-1.414)	0.884
Malignant ascites	1.970(1.518-2.557)	<0.001	1.199 (0.882-1.630)	0.246
Appendix	2.810(2.098-3.765)	<0.001	1.416 (0.983-2.093)	0.062
Primary treatment strategy	2.428 (1.867-3.156)	<0.001	1.786 (1.305-2.444)	<0.001
Colon except for rectosigmoid colon	2.510 (1.805-3.491)	<0.001	1.475 (1.000-2.177)	0.050
Upper Abdominal surgery	2.062(1.522-2.792)	<0.001	1.043 (0.674-1.6140	0.850
Omentum (cm)				
No	–	–	–	–
≤2	2.135 (1.507-3.024)	<0.001	1.044 (0.693-1.574)	0.835
>2	3.563 (2.635-4.817)	<0.001	1.393 (0.945-2.054)	0.094
Diaphragmatic top	2.914 (2.238-3.794)	<0.001	1.274 (0.871-1.862)	0.211
Liver surface	1.466 (1.023-2.100)	0.037	0.683 (0.449-1.040)	0.076
Liver parenchyma	2.146 (1.101-4.183)	0.025	1.657 (0.772-3.555)	0.195
Spleen	2.114 (1.339-3.337)	0.001	0.995 (0.573-1.727)	0.985
Pelvic floor tissue	1.659 (1.265-2.175)	<0.001	0.948 (0.693-1.297)	0.738
Bladder	2.349 (1.798-3.070)	<0.001	1.210 (0.873-1.676)	0.252
Small bowel and mesentery	2.590 (1.992-3.368)	<0.001	1.194 (0.858-1.661)	0.294
Residual tumor (cm)	3.348 (2.565-4.370)	<0.001	1.809 (1.284-2.548)	0.001
Chemotherapy cycle	0.582 (0.437-0.775)	<0.001	0.373 (0.267-0.522)	<0.001
Types of platinum	3.072 (2.023-4.665)	<0.001	1.884 (1.178-3.014)	0.008

In the lasso regression analysis, when the parameter λ was set to 0.047430, the models achieved a balance between complexity and performance. A total of seven variables were selected: types of platinum, FIGO stage, primary treatment strategy, appendix involvement, diaphragmatic top status, residual tumor size, and omentum condition.

Subsequently, utilizing these two sets of variables, we developed prediction models for platinum-resistance recurrence employing five machine learning algorithms. These models were then compared with traditional model fitting methods such as LR. We utilized the area under the curve (AUC) and average accuracy obtained through cross-validation as comprehensive measures of model performance. Additionally, we calculated sensitivity and specificity metrics to further evaluate our models.

#### 3.2.1 LR

##### 3.2.1.1 Based on the results of multivariate Logistic regression

**Figure 2:**
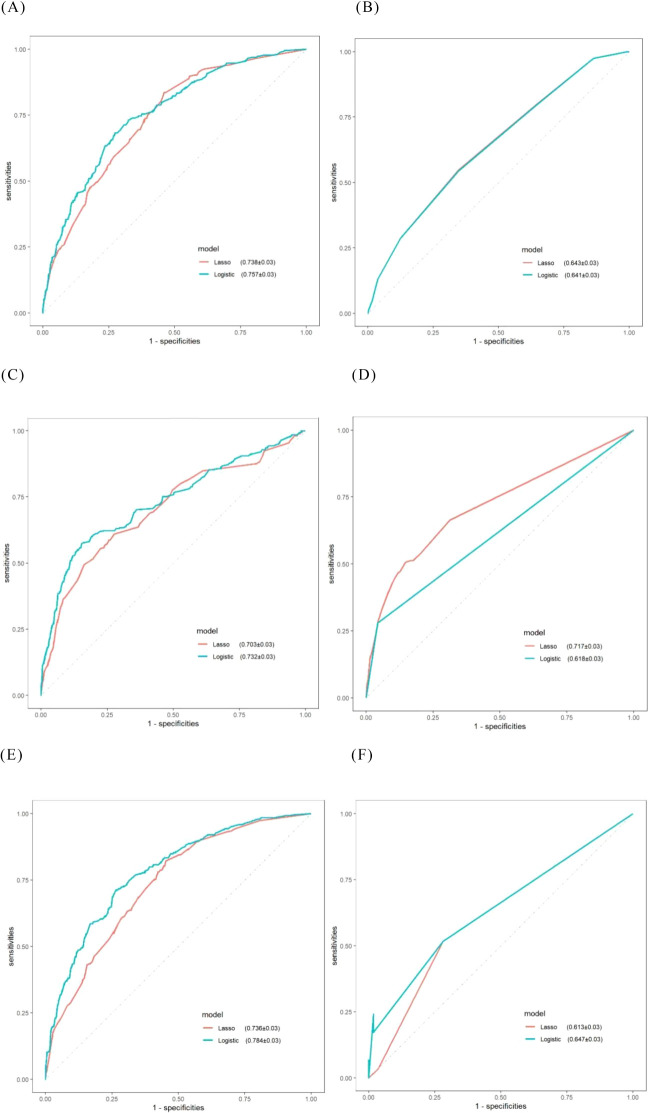
ROC curves for machine learning models obtained via internal validation. **(A)** LR; **(B)** KNN; **(C)** SVM; **(D)** RF; **(E)** XGBoost; **(F)** DTA.

Based on the results of multivariate logistic regression, the LR model established from the multivariate analysis yielded an AUC of 0.757 and an average accuracy of 81.3% (**Figure 2A**). This model will henceforth be referred to as “Logistic-LR.” The formula for predicting the probability of platinum resistance recurrence using the Logistic-LR model is: P= -5.59721 + 0.27335*X1 + 0.49898*X2 + 0.35923*X3 + 0.67842*X4 + 0.60799*X5 + 0.94185*X6 -0.18319 *X7*+0.81598 X8, X1 to X8 represent LDH, platelet count, FIGO stage, primary treatment strategy, supraclavicular lymph node metastasis, residual tumor size, chemotherapy cycle, and types of platinum respectively.

##### 3.2.1.2 Based on the results of lasso regression

**Figure 3:**
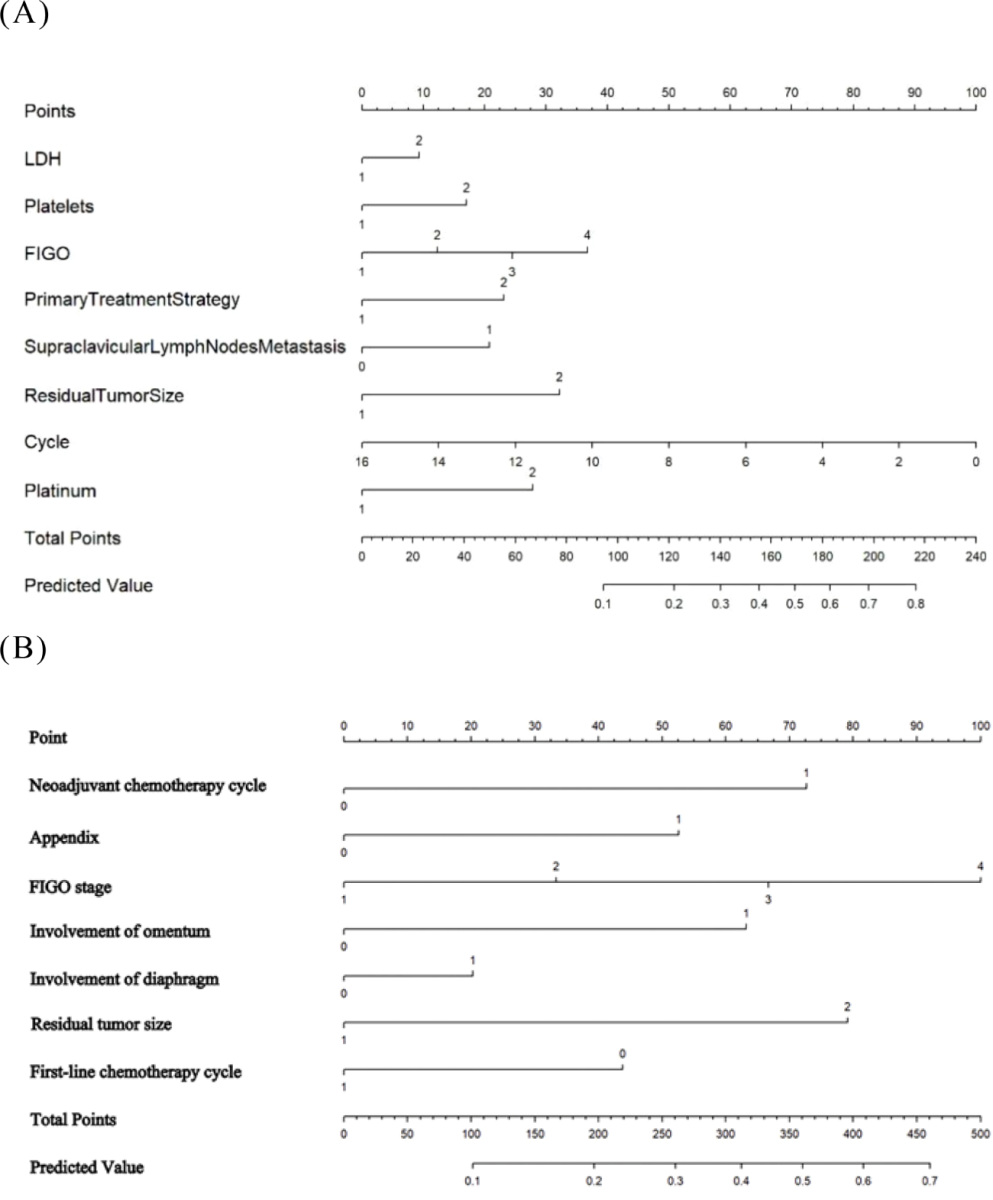
The developed nomogram predicting EOC platinum resistance recurrence. **(A)** The Logistic-LR model. **(B)** The Lasso- LR model.

The AUC for the LR model derived from lasso regression was found to be 0.738 (**Figure 2A**), with an average accuracy of 79.6%. This model will subsequently be referred to as “Lasso-LR.” Future models will follow this naming convention consistent with that used for LR models. The formula for predicting platinum resistance recurrence probability using the Lasso-LR model is: P= -4.8769 + 0.7401*X1 + 0.2255*X2 + 0.5815*X3 + 0.3846*X4 + 0.5730*X5 + 0.1573 *X6+ 0.5297 *X7, where X1 to X7 correspond to types of platinum, FIGO stage, primary treatment strategy, appendix, residual tumor size, diaphragmatic top and omentum respectively. Nomograms were constructed based on both LR models described above (**Figure 3**).

#### 3.2.2 KNN

##### 3.2.2.1 Based on the results of multivariate Logistic regression

The AUC of the “Logistic-KNN” model was 0.641 (**Figure 2B**). The cut-off value was established at 0.233, yielding a sensitivity of 0.653, specificity of 0.544, and an average accuracy of 67.0%.

##### 3.2.2.2 Based on the results of lasso regression

The AUC for the “Lasso-KNN” model was recorded at 0.643 (**Figure 2B**). At a cut-off value of 0.233, this model demonstrated a sensitivity of 0.653, specificity of 0.549, and an average accuracy of 66.1%.

#### 3.2.3 SVM

When employing SVM to construct models, we explored various kernels including linear kernel, RBF kernel, polynomial kernel, and sigmoid kernel respectively. Our analysis revealed that the RBF kernel provided the best fit for the model and exhibited strong performance in validation; Thus we ultimately selected it for our modeling approach.

##### 3.2.3.1 Based on the results of multivariate Logistic regression

The AUC for the “Logistic-SVM” model reached an impressive value of 0.732 (**Figure 2C**), with an average accuracy reported at 78.9%.

##### 3.2.3.2 Based on the results of lasso regression

The AUC for the “Lasso-SVM” model was determined to be 0.703 (**Figure 2C**), also achieving an average accuracy of approximately 78.9%.

#### 3.2.4 RF

##### 3.2.4.1 Based on the results of multivariate Logistic regression

The AUC of the “Logistic-RF” model was 0.618 (**Figure 2D**). At a cut-off value of 0.225, the sensitivity was recorded at 0.957, while the specificity stood at 0.279, resulting in an average accuracy of 79.2%.

##### 3.2.4.2 Based on the results of lasso regression

The AUC of the “Lasso-RF” model was 0.717 (**Figure 2D**), with an average accuracy of 79.0%. At a cut-off value of 0.075, the sensitivity was recorded at 0.853, while the specificity stood at 0.506.

**Figure 4:**
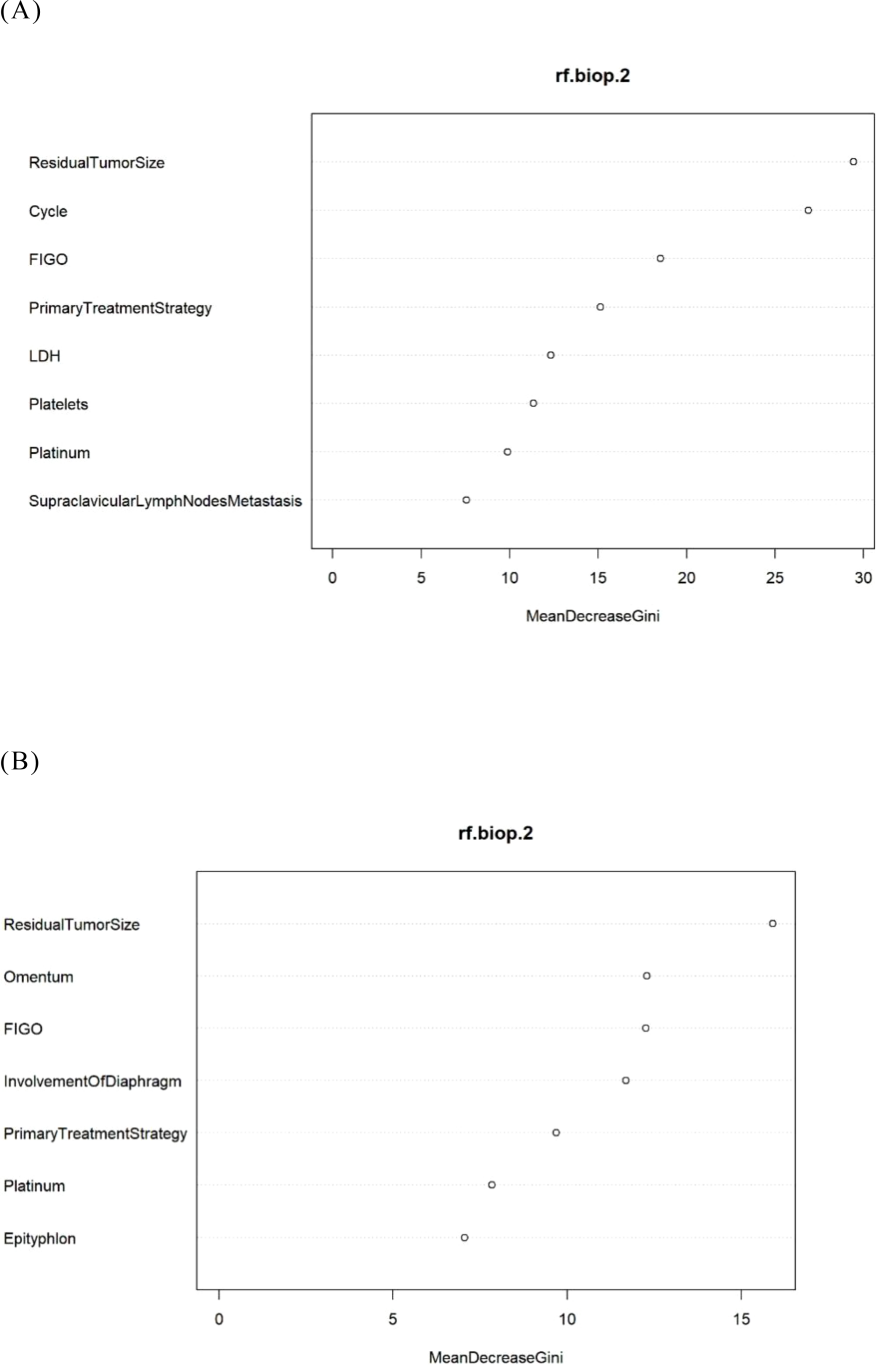
The feature importance in the RF model. **(A)** The Logistic-RF model. **(B)** The Lasso- RF model.

To enhance the transparency of the model, we presented the ranking of variable importance (26). The “MeanDecreaseGini” metric indicated each variable’s contribution—whether positive or negative—to the risk of platinum resistance recurrence as defined by the model (**Figures 4A, B**).

#### 3.2.5 XGBoost

##### 3.2.5.1 Based on the results of multivariate Logistic regression

The AUC for the “Logistic-XGBoost” model was 0.784 (see **Figure 2E**), with an average accuracy of 84.0%. At a cut-off value of 0.240, the sensitivity was recorded at 0.735 and the specificity at 0.713.

##### 3.2.5.2 Based on the results of lasso regression

The AUC of the “Lasso-XGBoost” model was 0.736 (**Figure 2E**), with an average accuracy of 79.2%. At a cut-off value of 0.330, the sensitivity was recorded at 0.548 and the specificity at 0.822.

**Figure 5:**
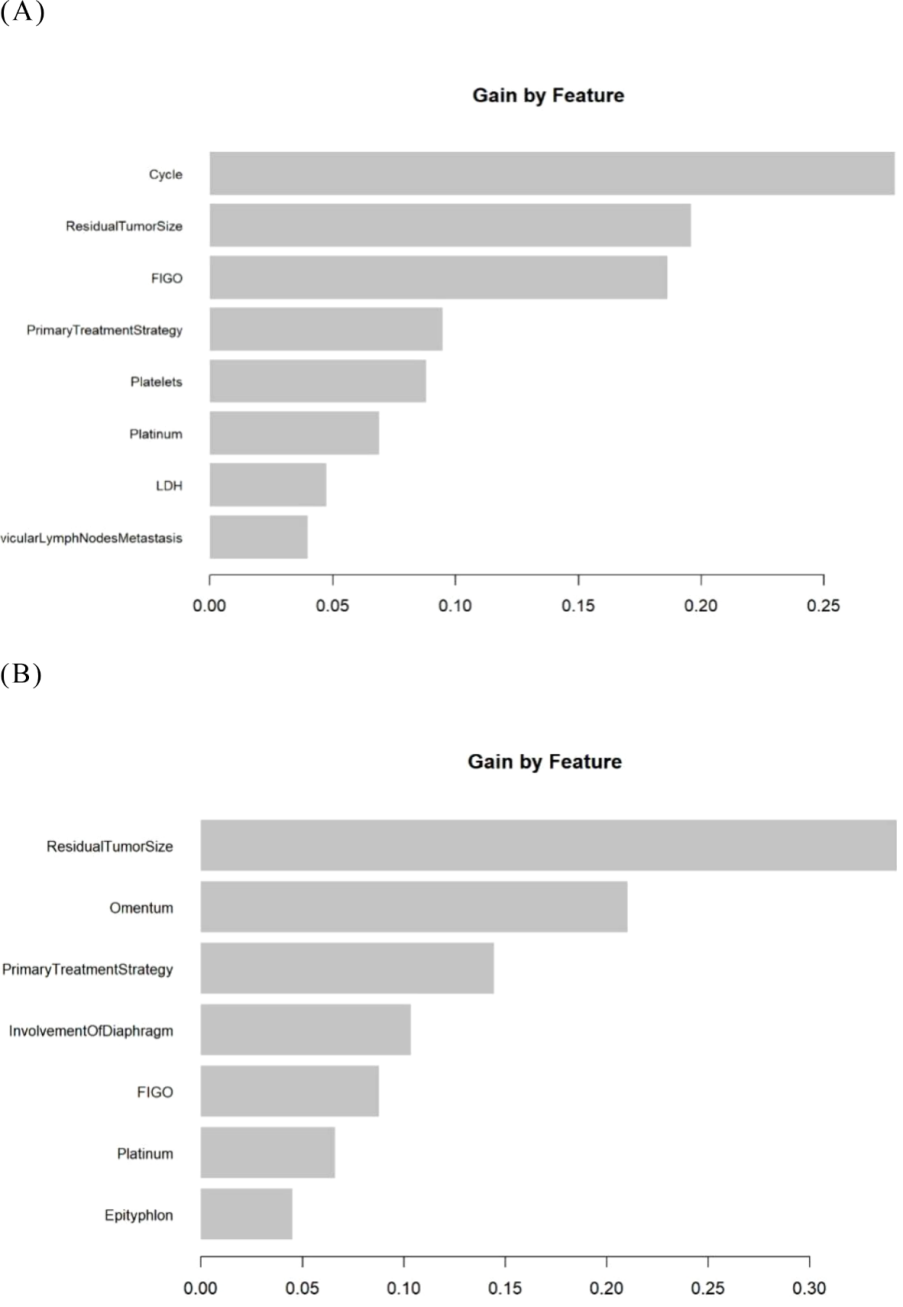
The importance ranking of variables in the XGBoost models. **(A)** The Logistic- XGBoost model. **(B)** The Lasso- XGBoost model.

In addition, we visualized the importance ranking of variables within the XGBoost model (**Figure 5**). Notably, the three most significant variables for predicting platinum resistance recurrence in the Logistic-XGBoost model were identified as chemotherapy cycle, residual tumor size, and FIGO stage.

#### 3.2.6 DTA

##### 3.2.6.1 Based on the results of multivariate Logistic regression

**Figure 6:**
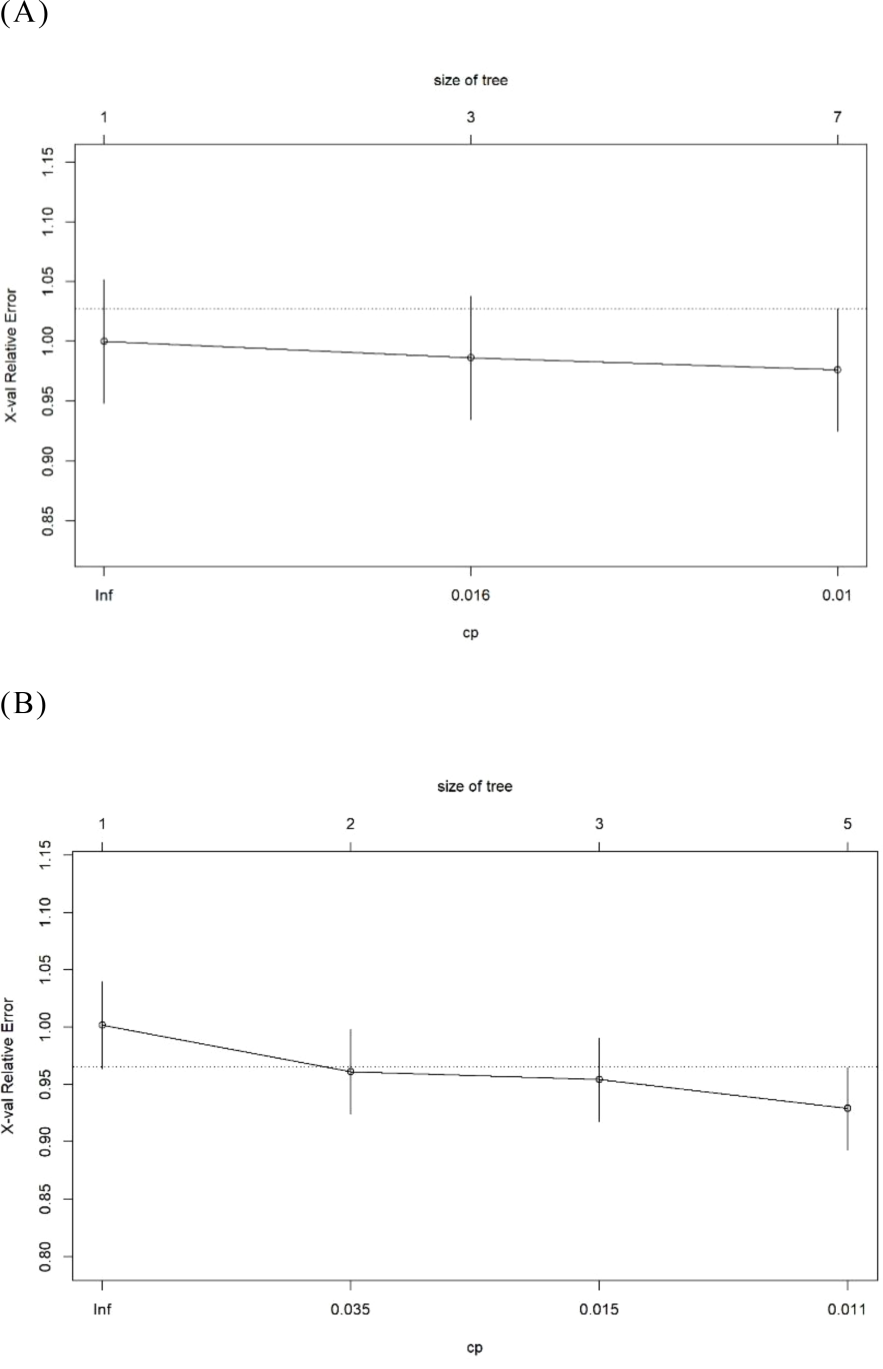
The goodness of fit graph in the DTA models. **(A)** The Logistic- DTA model. **(B)** The Lasso- DTA model.

The goodness-of-fit plot indicates that the optimal complexity parameter (cp) value was 0.01 (**Figure 6A**). We set the cp value to 0.01 and optimized the model through pruning. Ultimately, the AUC of the “Logistic-DTA” model reached 0.647, with an average accuracy of 79.3%. At a cut-off value of 0.217, the sensitivity was found to be 0.718, while the specificity was recorded at 0.517 (**Figure 2F**).

##### 3.2.6.2 Based on the results of lasso regression

The goodness-of-fit graph indicated that the optimal cp value was 0.011, which was subsequently set to this value (**Figure 6B**). Ultimately, the AUC of the “Lasso-DTA” model was determined to be 0.613, with an average accuracy of 78.2%. At a cut-off value of 0.239, the sensitivity reached 0.718 and specificity was recorded at 0.517.

**Figure 7:**
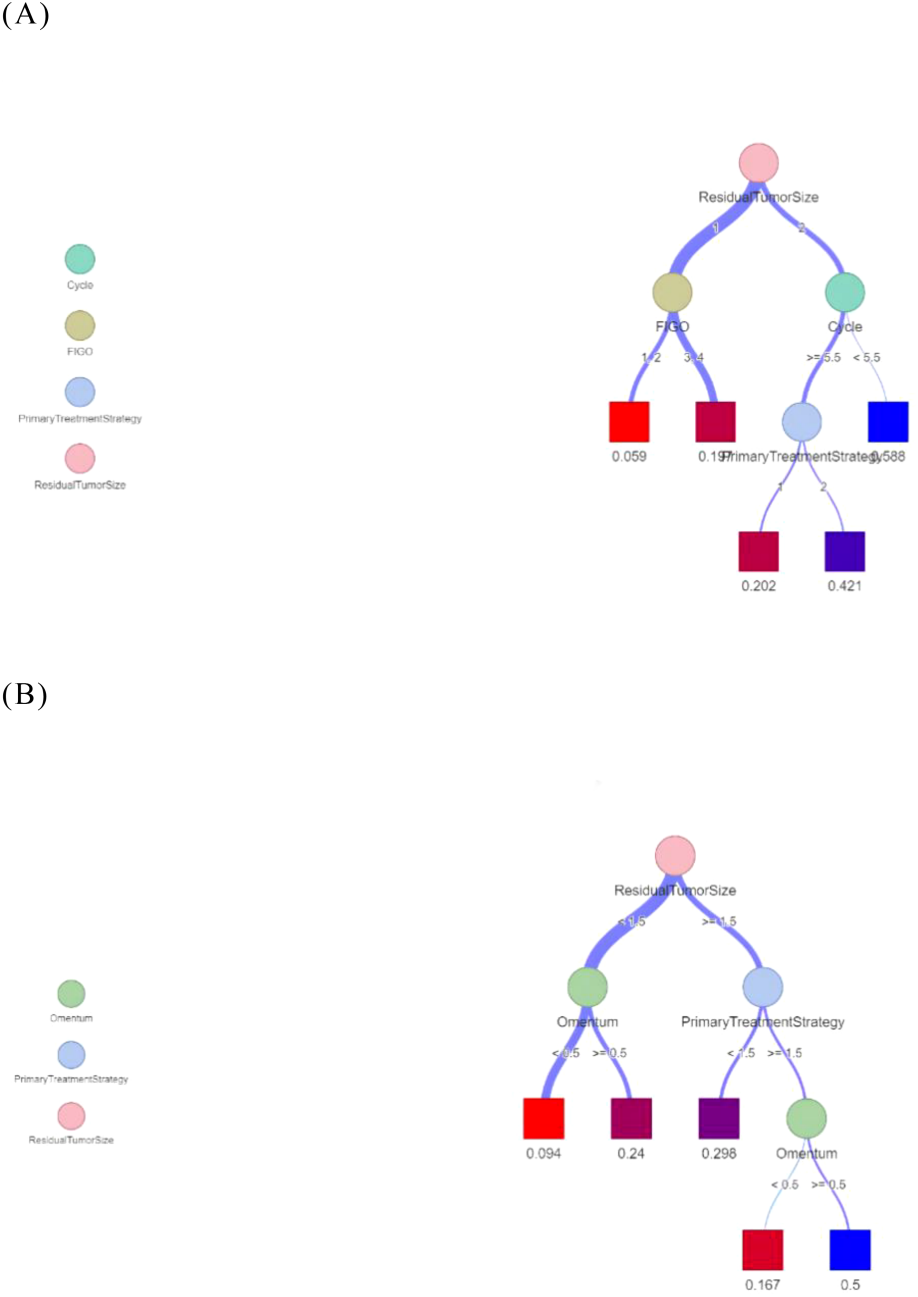
Decision trees for predicting recurrence of platinum resistance. **(A)** The Logistic- DTA model. **(B)** The Lasso- DTA model.

We visualized the decision tree model (**Figure 7**). The terminal boxes in the classification tree represented leaf nodes, each corresponding to the final probability of platinum resistance recurrence as derived from the decision tree analysis.

**Figure 8:**
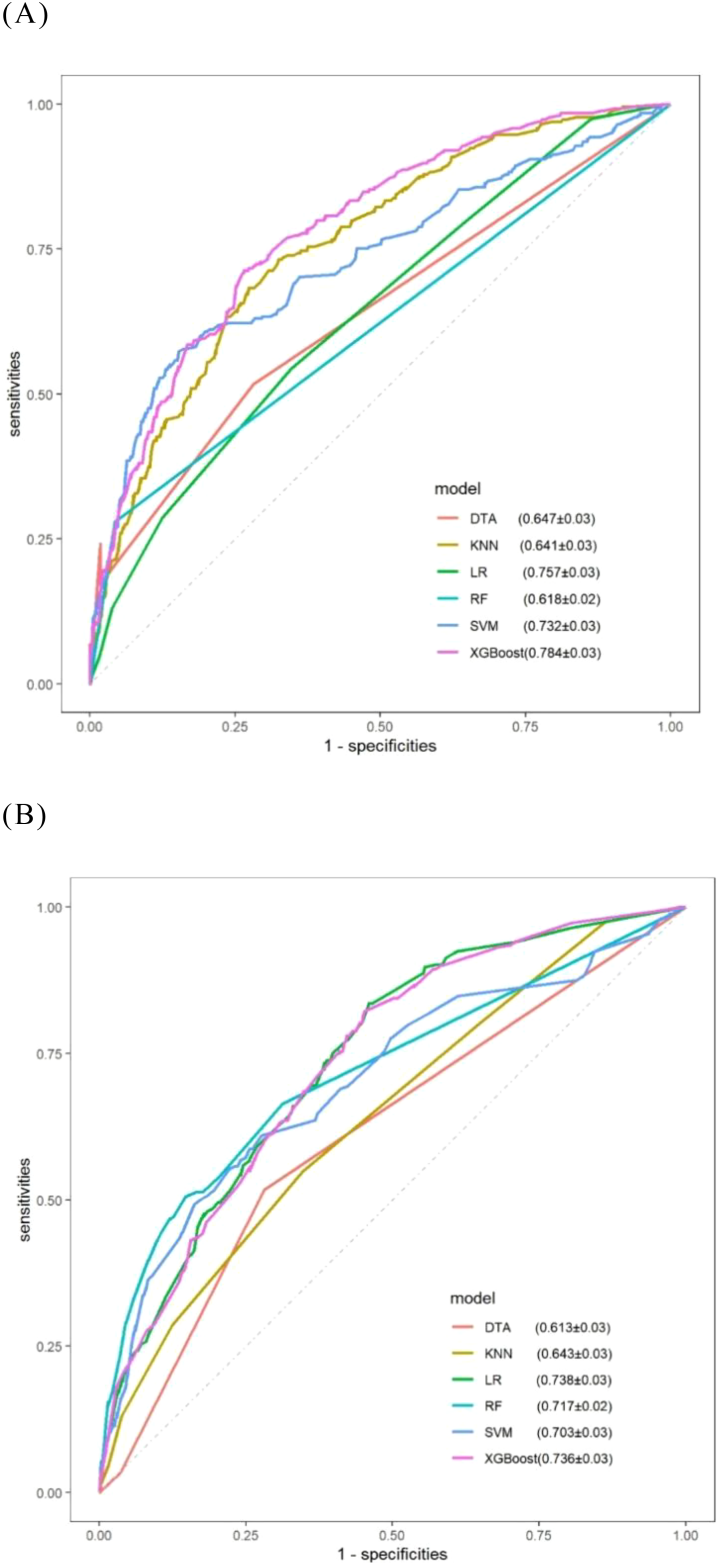
ROC curves for comparative models obtained via five-fold cross-validation. **(A)** Models consisting of eight selected variables; **(B)** Models consisting of seven selected variables.

**Table 3** illustrates the capability of each model to differentiate between platinum-sensitive and platinum-resistant cases by presenting specific metrics. The ROC curves for the six models, derived from five-fold cross-validation results, are displayed in **Figure 8**. The XGBoost model, which was developed based on eight variables identified through multivariate logistic regression analysis, demonstrated the highest performance among all models evaluated.

**Table 3:** Performance of machine learning models for platinum resistance recurrence.

Machine Learning		AUC	Sensitivity	Specificity	Balanced Accuracy	Threshold
LR	lasso	0.738	0.541	0.836	0.796	0.154
	logistic	0.757	0.727	0.683	0.798	0.221
KNN	lasso	0.643	0.653	0.549	0.661	0.233
	logistic	0.641	0.653	0.544	0.670	0.233
SVM	lasso	0.703	0.724	0.610	0.789	0.194
	logistic	0.732	0.847	0.574	0.789	0.194
RF	lasso	0.717	0.853	0.506	0.790	0.075
	logistic	0.618	0.957	0.279	0.792	0.500
XGBoost	lasso	0.736	0.548	0.822	0.792	0.330
	logistic	0.784	0.735	0.713	0.804	0.240
DTA	lasso	0.613	0.718	0.517	0.782	0.239
	logistic	0.647	0.718	0.517	0.793	0.217

## 4 Develop models based on previous literature and professional knowledge

In this study, the p-values for the appendix and omentum were found to be greater than 0.05 in the multivariate logistic regression analysis. However, several studies have identified the appendix and omentum as independent factors influencing platinum resistance recurrence. Additionally, results from lasso regression further indicated that both the appendix and omentum are significant independent predictors of platinum-resistant recurrence. Consequently, we adopted an exploratory approach to model development by integrating insights from existing literature with professional expertise. The appendix and omentum were incorporated either separately or simultaneously into eight variables selected through multivariate logistic regression to construct our model (**Table 4**). Notably, we observed a substantial enhancement in the performance of the RF model when both factors were included in its construction, with the AUC increasing from 0.627 to 0.887.

**Table 4:** Performance of machine learning models based on multivariate Logistic regression results and professional knowledge.

Variables	Machine Learning	AUC	Sensitivity	Specificity	Balanced Accuracy	Threshold
8 variables	LR	0.757	0.722	0.683	0.798	0.221
	KNN	0.641	0.653	0.544	0.663	0.233
	SVM	0.732	0.847	0.574	0.789	0.194
	RF	0.618	0.957	0.279	0.792	0.500
	XGBoost	0.784	0.735	0.713	0.804	0.240
	DTA	0.647	0.718	0.517	0.793	0.217
8 + omentum	LR	0.761	0.738	0.668	0.799	0.236
	KNN	0.642	0.653	0.544	0.668	0.223
	SVM	0.757	0.797	0.664	0.789	0.193
	RF	0.807	0.828	0.659	0.790	0.106
	XGBoost	0.763	0.577	0.815	0.797	0.331
	DTA	0.653	0.864	0.414	0.790	0.271
8+ omentum + appendix	LR	0.761	0.711	0.694	0.813	0.222
	KNN	0.642	0.653	0.544	0.670	0.233
	SVM	0.748	0.798	0.657	0.791	0.191
	RF	0.887	0.920	0.747	0.793	0.225
	XGBoost	0.789	0.816	0.623	0.810	0.286
	DTA	0.648	0.891	0.379	0.789	0.268

## 5 Discussion

The primary objective of this study is to develop a machine learning predictive model for assessing the risk of platinum-resistant recurrence in patients with EOC. Among the models evaluated, the Logistic-XGBoost exhibited superior performance (AUC = 0.784). We recommend utilizing the XGBoost model, which incorporates eight variables. This model can be implemented in clinical practice once pathological data are obtained following surgical treatment and is anticipated to contribute significantly to clinical trial design and future research endeavors.

The enhanced accuracy of the Logistic-XGBoost model underscores the significant contribution of hidden variables identified in previous studies, as well as the novel clinical relevance of these variables, including LDH (27) and surgery-related information. This highlights the potential utility of routine laboratory data and clinical indicators as biomarkers for platinum-resistant recurrence. Elevated serum LDH levels are observed in patients experiencing active tumor growth and tissue destruction. In recent years, extensive research has been conducted on the prognostic value of serum LDH across various cancers, including lymphoma, lung cancer, colorectal cancer, breast cancer, kidney cancer, and liver cancer (28).

Furthermore, combining serum LDH with other tumor markers such as alpha-fetoprotein, CA125, and human chorionic gonadotropin can enhance the accurate determination of histological types in ovarian cancer (29). This study presents a novel association between serum LDH levels and platinum-resistant recurrence in EOC, specifically indicating that higher LDH levels correlate with an increased likelihood of platinum-resistant recurrence. Future large-scale and rigorously designed prospective studies are essential to validate the clinical significance of these markers and establish precise cutoff values.

Previous studies have identified inflammatory factor indicators, such as white blood cell count, the neutrophil-to-lymphocyte ratio, and the platelet-to-lymphocyte ratio, as independent factors influencing platinum-resistant recurrence (30–32). However, during the preliminary data preprocessing of this study, it became evident that these ratios—including white blood cell count, absolute values of various white blood cell classifications, the neutrophil-to-lymphocyte ratio, and the platelet-to-lymphocyte ratio—do not serve as independent prognostic factors for platinum-resistant recurrence. It is crucial to acknowledge that systemic diseases unrelated to cancer (such as inflammatory conditions or infections) may affect peripheral blood complete counts and potentially lead to inaccuracies in analysis results.

In the model construction phase, this research utilized six distinct machine learning algorithms: LR, K-Nearest Neighbors KNN, RF, SVM, DTA, and XGBoost (33, 34). All of these are classified as supervised machine learning algorithms. Unlike unsupervised learning, which does not utilize labeled data, supervised learning provides the computer with labeled datasets for training and subsequently applies the acquired patterns to make predictions on unknown data. Each algorithm has its own unique strengths and weaknesses, leading to significantly varied performance when applied to the same dataset. Currently, no single algorithm can comprehensively solve all problems in this domain. Therefore, this study recommends that researchers conducting similar investigations consider employing all widely recognized algorithms in order to identify the most effective one for developing a clinical prediction model.

The potential advantages of machine learning include its capacity to detect complex patterns and greater flexibility in managing missing data, as well as accommodating nonlinear relationships among parameters. Notably, in this study, the model fitted using variable selection results from Lasso regression indicated that the traditional LR model performed optimally; at this stage, machine learning did not surpass traditional LR methods.

Furthermore, the black box nature of machine learning presents an additional limitation when employing these models. While traditional models can be articulated in a clear mathematical form such as f(x) = β0 + β1X1 + β2X2 + β3X3……, machine learning models resist such straightforward formulation, prioritizing predictive accuracy over interpretability. It is crucial to select an appropriate modeling approach based on the specific characteristics of the data and research objectives rather than adopting machine learning indiscriminately.

In constructing clinical prediction models, influencing factors may evolve due to temporal and spatial variations; thus, continuous adaptation of the model is necessary. This study provides a conceptual framework for model evolution.

This study acknowledges several limitations. Firstly, due to its retrospective design, this research may be subject to issues such as selection bias, information bias, confounding bias, and temporal bias. As a non-randomized observational study, it has the potential to either overestimate or underestimate the risk of platinum-resistant recurrence in ovarian cancer. Furthermore, the retrospective nature of the study makes missing or incomplete data in medical records an unavoidable challenge. For instance, there was a significant amount of missing data regarding pathological types (35), degrees of differentiation, and assessments of chemotherapy responses within this study. Some pathological types were classified merely as adenocarcinoma in the reports, which hindered further distinctions; consequently, histological type was not included in the variable selection. The rates of missing data for degree of differentiation and HE4 were 31.46% and 86.49%, respectively; thus, we excluded the analysis related to degree of differentiation. Given the extended time span involved in this research, time bias is inevitably present; future studies will require data from multi-center or large national databases to effectively evaluate the clinical applicability value of the model.

Secondly, while the model developed herein underwent internal validation only, it necessitates external validation across various timeframes and settings to enhance its applicability and generalizability further. Additionally, prospective studies are needed to more accurately assess its clinical utility and identify more precise variables for inclusion.

Lastly, currently only a subset of patients at our institution has undergone germline or somatic testing for breast cancer susceptibility genes (BRCA) 1/2; therefore, this study did not incorporate molecular characteristics such as gene mutations into variable considerations. As relevant genetic and molecular data accumulate at our institution, integrating results from BRCA1/2 gene testing with other molecular detection outcomes is anticipated to facilitate the development of a more accurate prediction model; furthermore, incorporating factors related to maintenance therapy should be considered in subsequent investigations.

Despite these limitations, this study has successfully established a prediction model for assessing platinum-resistant recurrence risk among EOC patients. With further validation and refinement, this model could enable early identification of patients at risk for platinum-resistant recurrence, ultimately improving prognosis for EOC patients.

## 6 Conclusions

1. Multiple logistic regression showed that LDH, FIGO stage, platelet count, supraclavicular lymph node metastasis, primary treatment strategy, residual tumor size, type of platinum (carboplatin/cisplatin or others), chemotherapy cycle were independent influencing factors of platinum resistance recurrence.

2. Among the constructed machine learning models, logistic-XGBoost model has the best performance, with an AUC of 0.784 and an average accuracy of 0.804. The model can be applied to clinical practice after obtaining pathological data in surgical treatment, and is expected to play a role in clinical trial design and future research.

With further refinement and external validation, the model can potentially improve the prognosis of EOC by early identification of platinum resistance recurrence.

3. When constructing the clinical prediction model, it is suggested that researchers try all the commonly used model fitting methods, and select the best fitting method that is most consistent with the data characteristics.

## Data Availability

The raw data supporting the conclusions of this article will be made available by the authors, without undue reservation.
